# Arthritis and Synovitis

**Published:** 1897-10

**Authors:** 


ARTHRITIS AND SYNOVITIS, BY DR. GUITTARD.—This author distinguishes two forms of this disease that are very grave. The one form due to the strain of the joint, and the other due to rheumatism. The first form, in the early stages of the disease, is best treated by means of cold applications; but when the acute inflammation has lessened it should be followed up by blistering. The author specially recommends an ointment consisting of the following: Bichromate potash, 4 grammes; iodide potash, 4 grammes; vaselin, 50 grammes.

In rheumatic arthritis of these parts it does not respond very quickly to treatment; the best means employed was the administration of 25 grammes of salicylate of soda per day, this to be administered for five or six days. Dr. Guittard claims that by the aid of this ointment he was successful in all his cases treated in this way when he got them in their initial state. When the case becomes semi-chronic they are very much more difficult to treat.—*Progrès Vétérinaire.*

